# Can Arthroplasty Stem INfluence Outcome? (CASINO): a randomized controlled equivalence trial of 125 mm versus 150 mm Exeter V40 stems in total hip arthroplasty

**DOI:** 10.1186/s13063-018-2621-8

**Published:** 2018-04-16

**Authors:** David F. Hamilton, Nicholas E. Ohly, Paul Gaston

**Affiliations:** 1 Department of Orthopaedics, University of Edinburgh, Chancellors Building, 49 Little France Crescent, Edinburgh, EH16 4SB UK; 2 Department of Orthopaedics, Golden Jubilee National Hospital, Clydebank, UK

**Keywords:** Total hip arthroplasty, Outcomes, Function

## Abstract

**Background:**

The use of shorter length femoral stems during total hip arthroplasty has been suggested to accommodate wider patient femoral geometry and offer maximal bone preservation. However, cemented short-stem designs may increase the risk of varus stem malalignment and influence patient outcomes.

**Methods/Design:**

CASINO is a multi-centre randomised equivalence trial that will recruit 220 patients undergoing total hip arthroplasty for osteoarthritis at two NHS hospitals in Scotland. Patients will be aged 45–80, undergoing unilateral primary hip arthroplasty, with no plan for contralateral procedure within the study timeframe, and able to comply with the protocol. Participants will be randomised to receive either a short (125 mm) or a standard (150 mm) Exeter V40 stem. The Contemporary acetabular component will be used in all cases. All implants will be cemented.

Patient pain, function and satisfaction will be assessed using change from baseline measurement in Oxford Hip Score, Forgotten Joint Score, EQ-5D, pain numerical rating scores, and patient satisfaction questionnaire at baseline and at 1 and 2 years following surgery. Radiographic assessment will evaluate stem position and will be appraised by independent reviewers. Patients will be blind to implant allocation.

**Discussion:**

Stem length may be associated with outcome; however, we can find no randomised trial in which researchers investigated the effect of stem length on patient outcome following cemented total hip arthroplasty. The aim of this trial is to determine if the use of short cemented stems offers equivalent patient outcomes to those achieved following surgery with standard length stems.

**Trial registration:**

International Standard Randomised Controlled Trial Number, ISRCTN13154542, Registered on 30 June 2017.

**Electronic supplementary material:**

The online version of this article (10.1186/s13063-018-2621-8) contains supplementary material, which is available to authorized users.

## Background

Total hip arthroplasty is one of the most successful and cost effective of all surgical procedures in terms of alleviating pain, improving quality of life and enhancing physical function [1–3]. Over 100,000 procedures were carried out in the UK in 2016, with established implants demonstrating 10-year survival rates of more than 95% [4]. Various companies produce implants for hip arthroplasty and most have good long-term track records of success. The orthopaedic manufacturing industry supports the continual development of implant technology with a view to enhancing patient outcomes and prolonging implant longevity.

Recent developments in hip arthroplasty have focused on achieving the best fit in all patients through the use of shorter femoral stems. Short stems are suggested to eliminate problems with proximal-distal mismatch, excessive femoral bowing and diaphyseal deformity, which can make the surgical intervention more challenging [5]. The implantation of smaller components hypothetically also results in less damage to bone and soft tissue, and offers preservation of bone stock, which is advantageous in the event that future revision surgery is required. Despite widespread use in clinical practice, there is a lack of randomised trial evidence to support the potential benefits of using the short stems in total hip arthroplasty [6].

The original Exeter total hip arthroplasty femoral stem was designed in 1970 at a length of 150 mm and has been employed successfully since. The Universal stem was introduced in 1988, with an identical implanted portion of the stem to the current design, but a different trunnion taper. The current ‘V40’ Exeter stem design with the V40 taper was introduced in 2001, with excellent reported long-term survivorship [4, 7]. Recently, however, it has become apparent that the design may not be optimal for all patients. The ‘original’ 150 mm stem may be difficult to insert in certain groups, such as Dorr A femurs and those with excessive bow or femoral deformity, e.g. from a previous fracture. This observation has led to the design of a shortened Exeter V40 stem (125 mm) for use in revision surgery with the exact same design characteristics aside from the shorter length. This shorter stem has been successfully employed for revision cases and is being increasingly used ‘off label’ in primary surgery, where the new geometry allows for a better anatomical fit in some cases. The worry is that this stem is too small and thus has too thin a cross section, with the potential of stem fracture occurring when using the revision stem in primary total hip replacement. As a result, the manufacturer has designed a range of 125 mm stem sizes that parallel the original 150 mm length stems for use in primary surgery. The new, shorter 125 mm stem range is sized at No. 1, thus providing better stem strength whilst being shorter to fit in Dorr A femurs. These ‘short’ stems have been CE marked and are in routine use. However, there are theoretical concerns that the shortened 125 mm stem length could result in an increased incidence of varus malalignment at the time of cementation, which in turn may be associated with worse patient clinical outcomes.

Choy et al. [8] recently used data from the Australian Orthopaedic Association National Joint Replacement Registry to suggest that, at 7 years post-surgery, there is no significant difference in the cumulative percent revision rate in the Exeter short stem (3.4%, 95% CI 2.4–4.8%) compared with the standard length Exeter stem (3.5%, 95% CI 3.3–3.8%); however, this data does not include patient quality of life and functional outcomes. Consequently, as part of the responsible introduction of implantable technology, a randomised trial comparing the new 125 mm stem design against standard 150 mm length stems should be conducted to ensure equivalence of outcomes compared to the original stem design.

## Methods

### Study aim

The primary objective of the ‘Can Arthroplasty Stem INfluence Outcome?’ (CASINO) study is to assess whether using the 125 mm Exeter V40 stem in total hip arthroplasty achieves an equivalent change in patient-reported pain and function as the 150 mm Exeter V40 stem at 1 year. Secondary aims are to evaluate comparative pre- to post-operative changes in joint-specific function, general health and health economic parameters.

### Design

CASINO is a multi-centre, single blind, randomised controlled equivalence trial assessing the influence of stem length in primary total hip arthroplasty. We are conducting this study under the guidance of the Consolidated Standards of Reporting Trials (CONSORT) Statement for randomized controlled trials. This paper is written according to the Standard Protocol Items: Recommendations for Interventional Trials (SPIRIT) 2013 Statement for reporting a clinical trial protocols [9] (Additional file 1).

CASINO is co-sponsored by the University of Edinburgh & NHS Lothian (ACCORD, The Queen’s Medical Research Institute, 47 Little France Crescent, Edinburgh, UK). Ethical approval was granted by the South East Scotland Research Ethics Committee 02 (reference: 16/SS/0176). The study is registered with International Standard Randomised Controlled Trial Number ISRCTN13154542.

### Study setting

The study will be performed at two high volume NHS orthopaedic units in Scotland, namely the Royal Infirmary of Edinburgh, Edinburgh, and at the Golden Jubilee National Hospital, Clydebank.

### Participants

All patients attending the routine NHS outpatient clinics of the trial surgeons that make a decision to undergo total hip arthroplasty and are listed for surgery will be screened for eligibility and, if suitable, invited to participate in the trial. Written informed consent will be obtained by a suitably qualified member of the research team at study entry.

Ineligible and non-recruited patients will undergo routine total hip arthroplasty adhering to the local policy and guidelines of the study centre.

### Eligibility criteria

#### Inclusion criteria

The inclusion criteria are patient ages 45–80, attending hospital for a planned primary total hip arthroplasty with standard implants for a diagnosis of osteoarthritis who are willing and able to comply with the study protocol.

#### Exclusion criteria

Exclusion criteria are dysplasia of the hip/acetabulum, requirement for acetabular bone grafting, planned bilateral procedures within the trial period, procedures performed exclusively for pain relief (such as for patients with no walking capacity) and patients with activity limiting pain in either knee or contralateral hip, which would confound the outcome assessments.

### Randomisation procedures

All eligible participants will be randomised on a 1:1 ratio to either a 125 or 150 mm Exeter V40 stem. Randomisation will be performed by a computer-generated number, stratified by centre using random block sizes. The study centre randomisation code is held by the local principal investigator, and consenting patients are accordingly allocated to treatment arm by the local research team. Implant group allocation is passed onto the clinical teams to coordinate surgery. Patients are blinded to the intervention arm.

Participants are free to withdraw from the study at any point or a participant can be withdrawn by the investigator. If withdrawal occurs, it will be documented on a participant ‘Change of Status’ Form. In the event of withdrawal, patients will be invited to provide final primary end-point data*.*

### Intervention

Patients will receive either an ‘original’ (150 mm) or ‘short’ (125 mm) Exeter stem as part of otherwise routine total hip arthroplasty at the study centres.

Procedures will be performed by consultant orthopaedic surgeons and their supervised trainees. A standard operative technique will be employed by all trial surgeons, using the posterior approach, Exeter V40 femoral component and Contemporary acetabular component (Stryker, Mahwah, New Jersey). All implants will be cemented. The routine post-operative patient care protocol of the study centre will be employed.

### Study outcomes and timelines

The trial primary endpoint is the comparative (between-group) change in Oxford Hip Score (OHS) from preoperation baseline to 1 year postoperation. Secondary outcomes are patient-reported outcome measures to evaluate pre- to postoperative changes in joint-specific function, pain, general health and health economic parameters. Radiographic evaluation will focus on stem position and cement mantle evaluation. Clinical complications (thrombosis, dislocation, infection and failure for any reason that results in reoperation) will be recorded.

#### Patient-reported outcomes

The OHS is a patient-reported outcome measure developed specifically to measure the impact of pain and functional disability on an individual’s life for the population of patients undergoing hip replacement [10]; as such, it is an extensively validated and widely adopted outcome measure in patients undergoing hip replacement surgery and is sensitive to detect changes over time.

The Forgotten Joint Score (FJS-12) is a patient-reported outcome scale to assess joint awareness in hips and knees during various activities of daily living. It uses a 5-point Likert response format, consists of 12 questions, and the raw score is transformed to a range from 0 to 100 points. High scores indicate good outcome, i.e. a high degree of being able to forget about the affected joint in daily life. The FJS has a low ceiling effect and especially discriminates between good, very good and excellent outcome after total hip or total knee arthroplasty. In its validation study, it showed high internal consistency and discriminated well between patient groups known to show different outcomes [11, 12].

The EQ-5D is a standardised instrument with five items for use as a measure of self-reported general health [13]. Applicable to a wide range of health conditions and treatments, it provides a simple descriptive profile and a single index value for health status. It is one of the most frequently used measures to gain quality of life scores for analysis in health economy as utility weights for calculating the quality-adjusted life years can be obtained.

Global hip pain severity will be assessed using an 11 point (0–10) numerical rating scale, where 0 represents no pain and 10 the worst possible pain. The validity and sensitivity of the numerical rating scale has been well documented [14]. As it has been suggested that using multiple measurements of pain status as opposed to a single value of ‘current pain’ may provide more realistic and meaningful measurements of pain intensity [15], separate assessments will be made of ‘worst pain’ and ‘perceived mean daily pain’ over the past week as has been specifically recommended for use in osteoarthritis clinical trials [16].

Patient satisfaction with the outcome of the study will be evaluated with a simple question as part of the questionnaire battery. A 5-point Likert scale response format will be employed with the options, namely very satisfied, satisfied, neither satisfied or dissatisfied, dissatisfied and very dissatisfied.

#### Radiographic evaluation

Clinical evaluation of radiographs will determine the implant position by assessing varus/valgus orientation on the antero-posterior film. The cement mantle will be evaluated as described by Barrack et al. [17]. Length at the hip will be assessed by evaluating a line drawn across the inferior aspect of the obturator foramen (‘tear drops’). Trial participant radiographs will be reviewed and graded by two independent experts (surgeons) from NHS Lothian. It is not possible to blind the reviewers to allocation (this will be apparent on the radiograph). The surgeons will review and report the films separately. In the event of disagreement, the surgeons will discuss the individual radiographs and reach a consensus opinion.

Femoral stem migration (in millimetres) will be assessed using computer-assisted Einzel-Bild-Roentgen Analyse-Femoral Component Analysis software and radiographs taken at each interval. This is a well validated method for measuring migration of total hip arthroplasty components using standard pelvic antero-posterior radiographs. A specificity of 100% and a sensitivity of 78% compared with Roentgen stereophotogrammetric analysis for the detection of migration of over 1 mm has been demonstrated [18].

#### Clinical complications

We will review for the known potential clinical complications associated with total hip replacement to ensure balance between trial arms. These known risks include deep vein thrombosis, dislocation, infection and failure for any reason that results in reoperation.

#### Harms

As the medical devices are CE marked for this intervention, there will be no formal adverse event reporting, however, clinical complications will be reviewed as described. If the investigator becomes aware of any serious unexpected adverse event or reaction, this will require expedited reporting to the sponsor by the investigator. Re-admission for elective surgery on a different joint does not constitute a serious adverse event or reaction. Protocol deviations will be recorded and any influence on outcomes incorporated into the study analysis.

#### Study assessment schedule

Informed consent, baseline demographics and initial pre-operative assessment will be performed at time of surgical pre-admission. Post-operative assessment will be performed at 1 year clinical review or by postal questionnaire; 2 year assessment will be by postal questionnaires. Study radiographs are those routinely performed as part of the clinical process during the hospital stay and at 1 year post-operative clinical review (Table 1).

**Table 1:** Patient visit schedule

Assessment	Pre-op	Inpatient	52 weeks	104 weeks
Patient consent^a^	X			
Baseline demographics	X			
Clinical outcomes				
Oxford Hip Score	X		X	X
Forgotten Joint Score	X		X	X
EQ-5D	X		X	X
Pain scores	X		X	X
Satisfaction questionnaire			X	X
Clinical compilations				
Complications questionnaire				X
Case note review				X
Radiographic outcomes^b^				
Antero-posterior pelvis	X	X	X	

^a^Consent taken prior to any research activity

^b^Radiographs taken as part of routine clinical practice. The initial post-operative radiograph is taken during the inpatient stay following the hip replacement

### Analysis

#### Sample size

The trial will be an equivalence study, powered on the change in the OHS that is considered clinically significant and of interest when comparing interventions (5 points) [19]. Data from the National Joint Registry indicates that patients typically report a change of 19.7 points (SD 10.4) on the OHS.

#### Power calculation

A total of 94 participants are required per group to detect a 5-point difference in the primary outcome between groups to achieve and alpha value of 0.05 and beta value of 0.9. Allowing for a potential 15% loss to follow-up we are targeting a total trial recruitment of 220 participants.

#### Statistical analysis

Analysis will be by intention to treat. Differences between groups will be estimated using the appropriate methodology depending on the distribution of data, either by *t* test or Wilcoxon test at the 5% significance level to compare the distribution of the primary endpoint between the treatment samples. The patient outcome scores will be assessed with repeated measures analysis of variance (ANOVA) models to account for the repeated data collection time points. Results will be presented as an adjusted mean difference with its corresponding 95% confidence intervals.

Should significant data volume be missing at random, we will incorporate a complete case analysis in addition to intention to treat. If there are concerns as to the balance of data in the trial arm, we will run sensitivity analyses. Subgroup analysis is planned by femur type (Dorr classification). Any further subgroup analysis will be post-hoc and clearly labelled as such. A data analysis plan will be finalised prior to data lock and the analysis will be conducted blind to intervention allocation.

### Data management

Each site will hold data according to the Data Protection Act 1998, and data will be collated in case report forms identified by a unique identification number only. Recruitment logs at the sites will list the identification numbers. All data recorded electronically will be held on password-protected NHS trust information technology systems with permission for access as detailed in the delegation log. All study files will be stored in accordance with Good Clinical Practice guidelines. Study documents held by the sponsor will be retained in a secure, locked location for the duration of the trial. All essential documents, including source documents, will be retained for a minimum period of 5 years after study completion. All work will be conducted following NHS trust data protection policy.

In order to eliminate possible data entry errors, individual data will be compared to a range of plausible values. After data entry, random checks will be performed to search for internal inconsistencies, range errors or missing data. The assessors and data entry personnel will undergo training prior to the start of CASINO.

### Monitoring

Investigators and institutions involved in the study will permit trial-related monitoring and audits on behalf of the sponsor, Research Ethics Committee review and regulatory inspection(s). In the event of an audit or monitoring, the Investigator agrees to allow the representatives of the sponsor direct access to all study records and source documentation. In the event of regulatory inspection, the Investigator agrees to allow inspectors direct access to all study records and source documentation.

### Protocol amendments

Amendments to the protocol must be submitted in writing to the appropriate Research Ethics Committee, Regulatory Authority and local research and development for approval prior to participants being enrolled into an amended protocol.

## Discussion

The primary aim of CASINO is to evaluate whether differences in the length of femoral stem used results in differences in patient outcomes following total hip arthroplasty. The Exeter V40 is the dominant brand of cemented femoral stem used in the UK with greater than 60% of market share [20]. The stem range has been expanded to include short stem options. Whilst national joint registries collect data regarding the implantable devices, the granularity of implant data is variable and few routinely collate patient outcome scores. To our knowledge, there is no randomized trial evidence to evaluate whether the outcome of 125 mm short stem procedures are equivalent to those following procedures carried with the original 150 mm stem length.

### Strengths and limitations

CASINO benefits from the use of a variety of outcome metrics. We are collecting the OHS and EQ-5D as these are the mainstay of outcome analysis following total hip arthroplasty in the UK and this facilitates wider comparative interpretation. These questionnaires are augmented by the use of more sensitive and discriminative metrics. The FJS-12 has proven to be a more responsive tool, with greater measurement range, improved discriminative ability and effect size compared to the OHS [21]. Pain intensity is evaluated with direct evaluation of that construct using multiple rating scales. Radiographic review will directly evaluate any difference in position of the stem.

Patient outcome metrics can be influenced by dysfunction in the contralateral limb, and as such  we aim to ensure that the patients taking part in CASINO are not suffering symptomatic bilateral joint disease that would likely result in both hips being replaced within the study timeframe. This inclusion criteria enhances the validity of the study outcomes; however, it may compromise the generalisability of the findings. CASINO has been designed to investigate short-term clinical and patient-reported outcomes, however, the longer term metric of the success of an implant is longevity and surgical revision rate. 95% implant survival at 10 years is expected of all modern prostheses and such evaluation is outside the scope of this study. However, to facilitate future longer term evaluation, of this study cohort we have caped the age of participants to CASINO at 80 years. This inclusion criterion many also somewhat limit generalisability.

The results of this trial will be presented at scientific meetings and submitted for publication in relevant peer-reviewed journals. Results will be made available to patients on the public facing website of the trial centres. Published results will not contain any patient-identifiable data and will be presented for the whole group rather than for individual participants.

## Trial status

CASINO opened to recruitment on the July 1, 2017. Data collection is ongoing with an anticipated recruitment end date of June 29, 2018.

## Additional file

Additional file 1: SPIRIT checklist. (PDF 236 kb)

## Abbreviations

CASINO: Can Arthroplasty Stem INfluence Outcome?
FJS: Forgotten Joint Score
OHS: Oxford Hip Score
